# Porto‐sinusoidal vascular disorder in a pediatric patient with prolidase deficiency: A case report

**DOI:** 10.1002/jpr3.70055

**Published:** 2025-06-19

**Authors:** Melissa Castro, Christian Martinez, Carole Brathwaite, Reuven Bromberg, Ana M. Rodriguez, Erick Hernandez

**Affiliations:** ^1^ Division of Pediatric Gastroenterology, Hepatology, and Nutrition Vanderbilt University Medical Center Nashville Tennessee USA; ^2^ Department of Pediatrics Nicklaus Children's Hospital Miami Florida USA; ^3^ Department of Pathology and Laboratory Medicine Nicklaus Children's Hospital Miami Florida USA; ^4^ Division of Pediatric Rheumatology Nicklaus Children's Hospital Miami Florida USA; ^5^ Division of Clinical Genetics & Metabolism Nicklaus Children's Hospital Miami Florida USA; ^6^ Division of Pediatric Gastroenterology, Hepatology, and Nutrition Nicklaus Children's Hospital Miami Florida USA

**Keywords:** IL‐6 monoclonal antibody, nodular regenerative hyperplasia, portal hypertension, vasculopathies

## Abstract

Prolidase deficiency (PD) is a rare autosomal recessive disorder affecting collagen turnover, leading to diverse clinical manifestations including dermatologic lesions, hepatosplenomegaly, and vascular anomalies. Liver involvement in PD is poorly understood, with few reported cases. We present a child with early‐onset non‐cirrhotic portal hypertension and PD. The patient initially presented with neonatal hemolytic anemia and hepatosplenomegaly. At age 9, recurrent epistaxis and splenomegaly led to splenectomy. Liver biopsy revealed sinusoidal dilation and parenchymal nodularity, later progressing to esophageal varices. Genetic testing identified pathogenic variants in peptidase‐D gene, suggestive of PD, and biochemical testing confirmed the diagnosis. Given suspected vasculopathy, tocilizumab was initiated with clinical improvement. This case suggests a potential link between PD and porto‐sinusoidal vascular disorder (PSVD), particularly nodular regenerative hyperplasia. Further research is needed to explore prolidase's role in vascular remodeling and its contribution to PSVD‐related liver pathology. Early recognition may improve management and outcomes.

## 1 INTRODUCTION

Prolidase deficiency (PD) is a rare autosomal recessive disorder caused by a mutation in the peptidase‐D (*PEPD*) gene that leads to reduced prolidase enzyme activity. Prolidase is a dipeptidase that catalyzes the rate‐limiting step during collagen turnover by cleaving imidodipeptides containing C‐terminal proline and hydroxyproline.^1^, ^2^ Deficit of this enzyme results in increased urinary excretion of proline and hydroxyproline, therefore impeding recycling of proline for collagen synthesis, the main component of the extracellular matrix.^2^ Clinical manifestations vary and include skin lesions, hepatomegaly with elevated liver enzymes, splenomegaly, hematological anomalies, dysmorphic facial features, intellectual disability, and vascular anomalies.^1^ Dermatological involvement is the most common manifestation, characterized by chronic and recurrent ulcerations, predominantly in the lower limbs, which are resistant to treatment. Although liver involvement has been described, very few cases have been reported. The diagnosis of PD is challenging as symptoms appear progressively between the neonatal period and adulthood.^1^ Here, we present the case of a child with porto‐sinusoidal vascular disorder (PSVD) with portal hypertension (PH) and PD.

## 2 CASE REPORT

At birth, the patient presented with hemolytic anemia and hepatosplenomegaly with elevated liver enzymes, pseudo‐thrombocytopenia, and spherocytosis. Extensive gastrointestinal, hematological, and infectious workup were unremarkable. He was asymptomatic until age 9 when he presented with recurrent epistaxis and splenomegaly measuring 13.1 cm in craniocaudal dimension on abdominal ultrasound. Platelets were 58 × 10^3^/µL and ultimately underwent splenectomy. Portal ultrasound showed no portosystemic collateral vessels with normal caliber of vessels.

**Figure 1:**
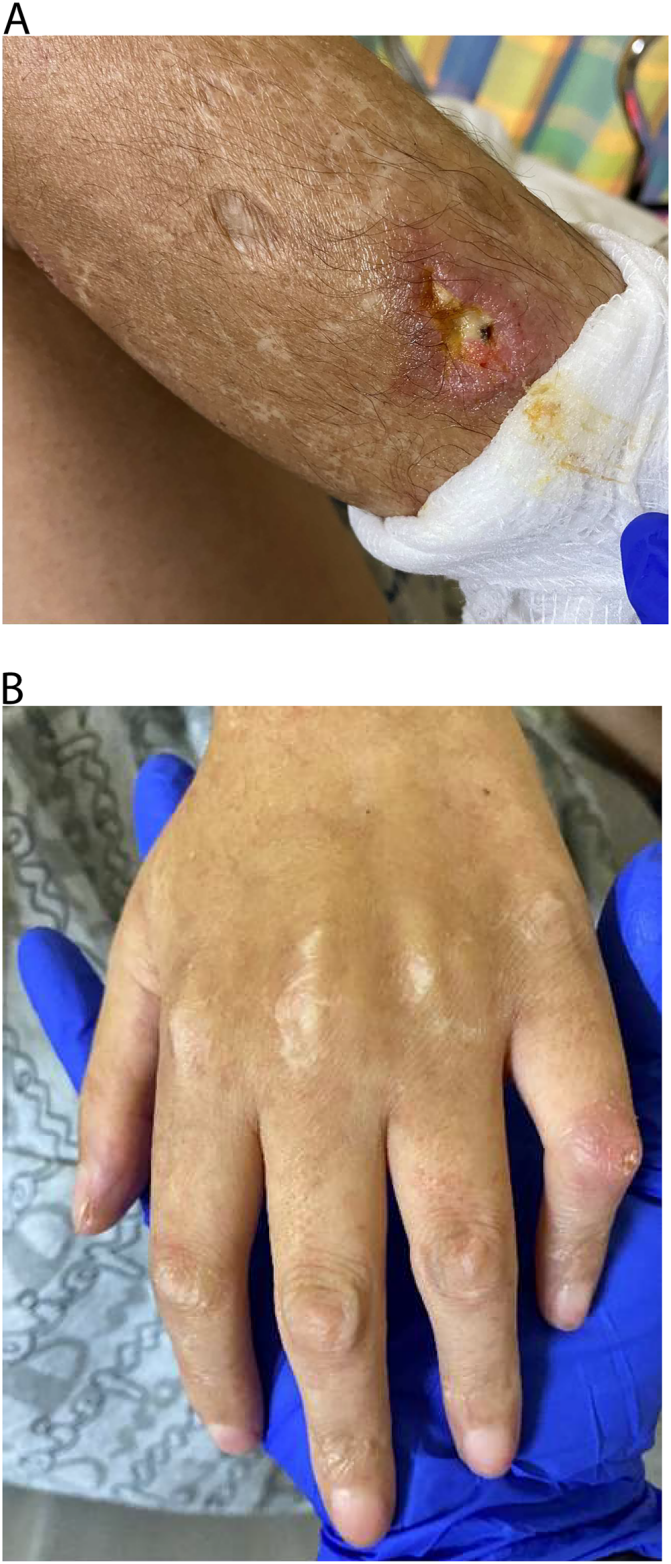
Skin lesions. (A) Ulcerated lesion over elbow. (B) Taught skin with prominent middle phalanges.

Due to persistent hepatitis, he underwent workup by a gastroenterologist at the age of 10. Liver biopsy revealed marked sinusoidal dilation with minimal inflammatory changes and without fibrosis. Autoimmune hepatitis, autoimmune vasculitis, a metabolic liver disease, and underlying cardiopathy were considered as possible diagnosis. He subsequently developed a skin rash with tender, tense, and nonhealing ulcers (Figure 1A,B). He was evaluated by cardiology, rheumatology, genetics, and hematology. Rheumatologic workup revealed elevated creatinine kinase: 10,440 IU/L, lactate dehydrogenase: 4392 IU/L, alanine transaminase/aspartate aminotransferase: 216/776 IU/L, and immunoglobulin G at 2506 mg/dL; albumin: 3.9 gm/dL, prothrombin time/international normalized ratio: 14.9 s/1.15, and positive for ribonucleoprotein, anti‐Sjögren's‐syndrome‐related antigen A antibody, smooth muscle, chromatin antibodies, and ANA. Normal chromosomal microarray and liver‐kidney microsome antibodies. Echocardiogram, serum ceruloplasmin level, 24‐h urine copper, and liver cholestatic panel by Prevention Genetics were all normal. Based on the workup and clinical presentation, the rheumatologist diagnosed the patient with mixed connective tissue disorder (MCTD) as the most likely diagnosis and was started on mycophenolate mofetil.

**Figure 2:**
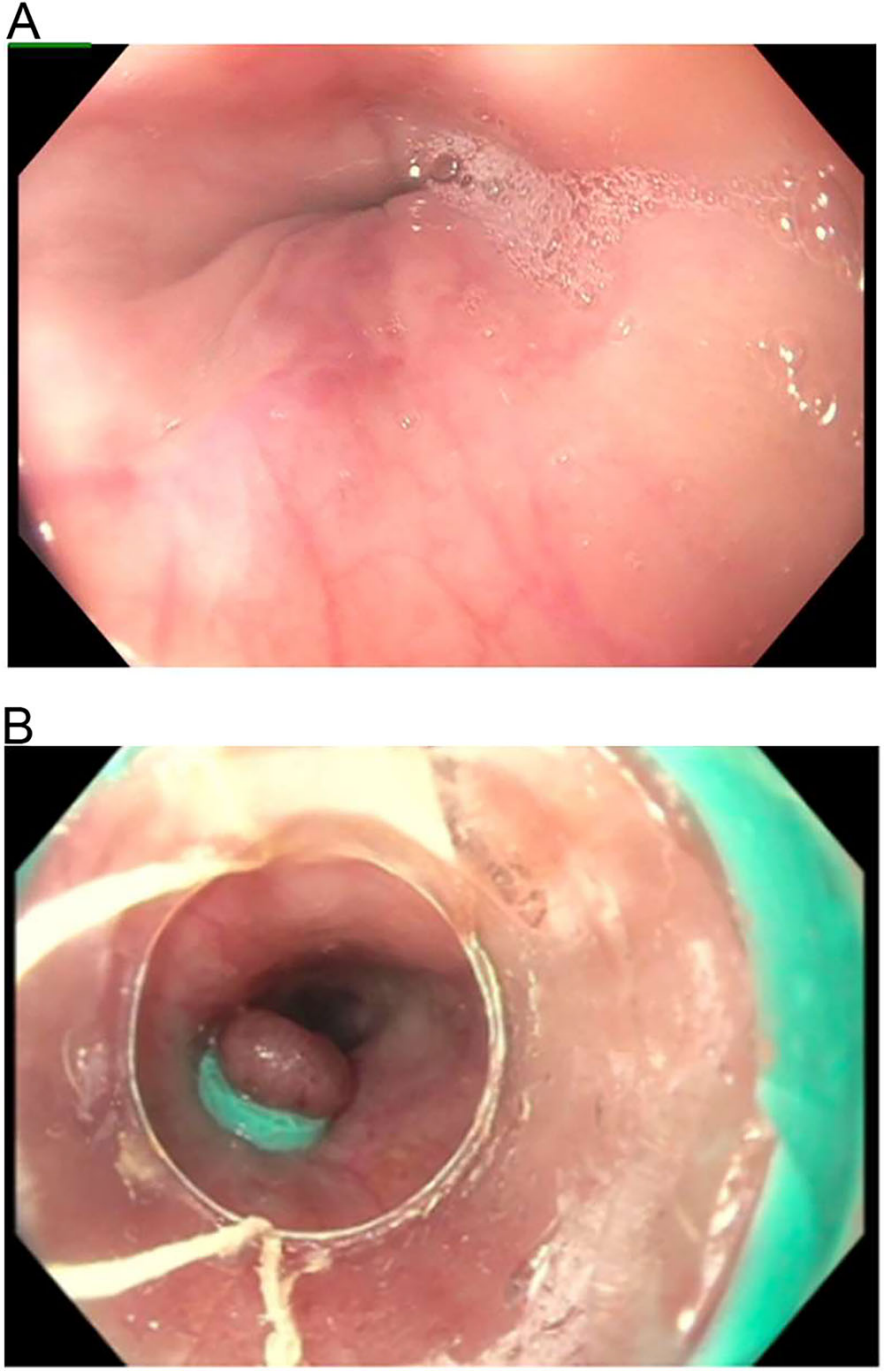
Endoscopic esophageal findings. (A) Grade 1 esophageal varices in the distal esophagus. (B) Esophageal band placement.

**Figure 3:**
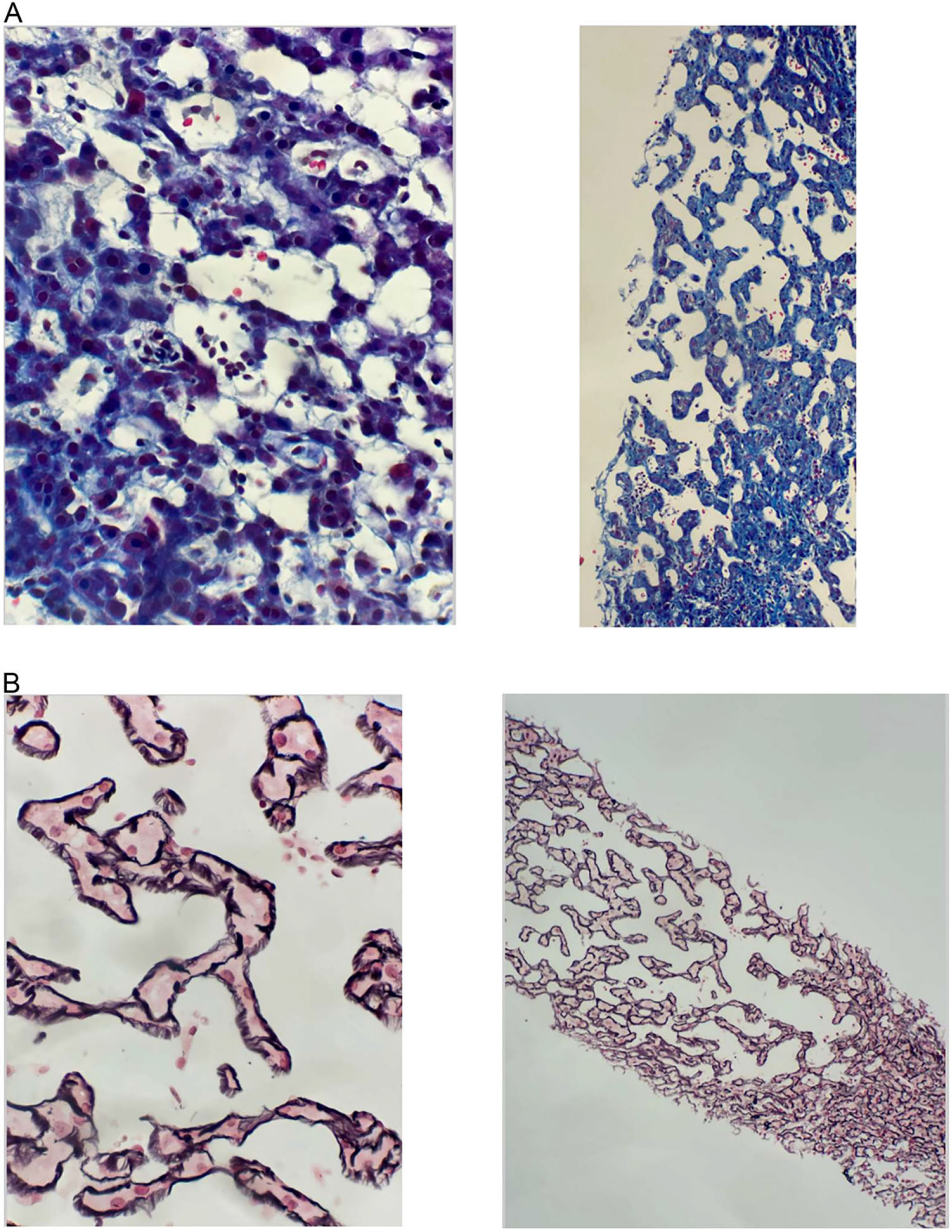
Histologic liver findings. (A) High (left) and low (right) power of liver biopsy with Trichrome staining. (B) High (left) and low (right) power of liver biopsy with reticulin staining.

He was lost to follow up with gastroenterology and returned 1 year later, hemodynamically stable, with upper gastrointestinal bleeding and anemia. Endoscopy revealed grade one esophageal varices with no active bleeding. Banding was performed (Figure 2A,B). High‐dose proton pump inhibitors and beta‐blockers were administered. A repeat liver biopsy showed worsening sinusoidal dilation and negative trichrome and reticulin staining with minimal to no inflammatory changes (Figure 3A,B). Portal Doppler US, liver magnetic resonance imaging (MRI), and MRI angiogram of the liver did not show signs of thrombosis but marked liver nodularity. He was examined by another rheumatologist who ultimately diagnosed him with MCTD with scleroderma and juvenile dermatomyositis overlap. Genome sequencing was repeated and a pathogenic heterozygous c.671+2 T > G variant and two variants of uncertain significance, c. 1354 G > A (p.Glu452Lys), and c.671+3_671+11del (Intronic), were identified in the *PEPD* gene on Invitae Inborn Errors of Immunity and Cytopenia Panel, associated with PD (Table S1). Proline urine acid analysis revealed 16 nmol/mg Cr and sample hydrolysis analysis revealed 4926 nmol/mg Cr, consistent with PD. He is currently being treated by a rheumatologist with tocilizumab, an interleukin (IL)‐6 inhibiting monoclonal antibody, with symptom improvement.

## 3 DISCUSSION

PSVD includes a group of vascular diseases of the liver characterized by specific histological findings such as nodular regenerative hyperplasia (NRH), obliterative portal venopathy, and incomplete septal fibrosis/cirrhosis, irrespective of the presence or absence of PH.^3^ NRH represents a key histologic variant of PSVD, characterized by diffuse micronodularity of the liver parenchyma without fibrosis, which may result from sinusoidal ischemia and vascular remodeling.^3^ Recognition of NRH as part of the PSVD spectrum underscores the importance of vascular alterations in the pathogenesis of non‐cirrhotic PH.^3^ Vasculopathies lead to increased resistance to portal blood flow due to either obstruction or distortion of the intra‐ or extra‐hepatic vasculature. NRH represents an example of distorted hepatic vasculature contributing to PSVD.^4^, ^5^ Given that PSVD may develop in the absence of classical risk factors for chronic liver disease, its pathogenesis likely involves complex interactions between immune dysregulation, endothelial dysfunction, and genetic susceptibility.^3^

Following the initial liver biopsy, the differential diagnoses included autoimmune hepatitis, vasculitis, a metabolic, and a cardiovascular process. However, additional workup excluded these conditions, and histological and clinical findings pointed toward an underlying vasculopathy, most likely associated with MCTD. Although PSVD was not initially suspected, subsequent findings aligned with this diagnosis. We propose that prolidase dysfunction may have contributed to vascular remodeling and sinusoidal injury underlying PSVD. As described, prolidase is crucial for extracellular matrix remodeling, degrading collagen imidodipeptides, and recycling proline for collagen synthesis.^2^ Deficiency in prolidase disrupts collagen turnover and may impair vascular remodeling, potentially exacerbating sinusoidal damage and contributing to PSVD‐like pathology. While the direct role of PD in liver disease remains unclear, collagen dysregulation may play a role in predisposing to or amplifying vascular injury associated with PSVD. To our knowledge, no cases of PD diagnosed in childhood have been associated with PSVD with PH.^6^, ^7^ Given the suspected vasculopathy, the patient was initiated on tocilizumab, an IL‐6 inhibiting monoclonal antibody. IL‐6 plays a role in multiple biological processes, including inflammation and immune regulation, and is implicated in the pathogenesis of antineutrophil cytoplasmic antibody‐associated vasculitis.^8^ Following treatment, the patient demonstrated significant clinical improvement, including resolution of esophageal varices, near‐complete resolution of skin findings, and discontinuation of propranolol.

## 4 CONCLUSION

Determining the etiology of PSVD is critical for guiding management and preventing complications such as esophageal variceal bleeding. Identification of additional genetic and immunological factors implicated in liver disease may enable earlier recognition of at‐risk individuals. Future research into the molecular mechanisms underlying PSVD and its overlap with metabolic and genetic disorders, such as PD, may provide novel therapeutic targets.

## CONFLICT OF INTEREST STATEMENT

The authors declare no conflict of interest.

## ETHICS STATEMENT

Written informed consent was obtained from the patient's legal guardian for publication of this case report and any accompanying images. The case was reviewed in accordance with institutional policies, and all identifying information has been omitted to maintain patient confidentiality.

## Supporting information

Supporting information.
