# Supplementary material for: Porto‐sinusoidal vascular disorder in a pediatric patient with prolidase deficiency: A case report
Source: JPGN Rep. 2025 Jun 19;6(4):519–23. doi: 10.1002/jpr3.70055 (PMC12611586; doi:10.1002/jpr3.70055)
Supplement: Supplementary file 1 — Supporting information. [file JPR3-6-519-s001.docx]

**Supplemental Table S1. Genetic Variants Associated with Prolidase Deficiency**

| **Gene** | ***PEPD*** | ***PEPD*** | ***PEPD*** |
| --- | --- | --- | --- |
| **Variant** | c.671+2T>G (Splice donor) | c.1354G>A; p.Glu452Lys | c.671+3_671+11del (Intronic) |
| **ClinVar** | NM_000285.4 | NM_000285.4 | NM_000285.4 |
| **Genomic location** | Intron 9 | Exon 15 | Intron 9 |
| **Zygosity** | Heterozygous | Heterozygous | Heterozygous |
| **Segregation** | Maternally inherited | Paternally inherited | Maternally inherited |
| **Variant classification** | Likely pathogenic | Uncertain significance | Uncertain significance |
| **Functional Prediction Studies** | Loss of function by disruption of RNA splicing. Loss of function is a known mechanism of disease | Missense change by replacing glutamic acid (acidic and polar) with Lysine (basic and polar). Predictive algorithms on the effects of missense changes in protein function and structure are inconclusive | Affects one nucleotide within the consensus splice site. However, it does not directly change the encoded amino acid sequence.  Predictive algorithms suggest that this variant might cause aberrant splicing. |
| **Population databases** | Not present | rs767937361, gnomAD 0.1% | Not present |

*PEPD:* peptidase-D gene
